# Towards Achilles Tendon Injury Prevention in Athletes with Structural MRI Biomarkers: A Machine Learning Approach

**DOI:** 10.1186/s40798-024-00786-6

**Published:** 2024-11-05

**Authors:** Norbert Kapinski, Karol Jaskulski, Justyna Witkowska, Adam Kozlowski, Pawel Adamczyk, Bartosz Wysoczanski, Agnieszka Zdrodowska, Adam Niemaszyk, Beata Ciszkowska-Lyson, Michal Starczewski

**Affiliations:** 1 Interdisciplinary Centre for Mathematical and Computational Modelling, University of Warsaw, Warsaw, Poland; 2 Smarter Diagnostics, Olsztyn, Poland; 3 Faculty of Rehabilitation, Jozef Pilsudski University of Physical Education in Warsaw, Warsaw, Poland; 4 Faculty of Materials Science and Engineering, Warsaw University of Technology, Warsaw, Poland; 5 Gamma Medical Center, Warsaw, Poland; 6 MIRAI Clinic, Otwock, Poland

**Keywords:** Achilles tendon, Injury prevention, Artificial intelligence, Magnetic resonance imaging

## Abstract

**Background:**

Recent advancements in artificial intelligence have proven their effectiveness in orthopaedic settings, especially in tasks like medical image analysis. This study compares human musculoskeletal radiologists to artificial intelligence in a novel, detailed, short, and cost-effective examination of Achilles tendon magnetic resonance images to uncover potential disparities in their reasoning approaches. Aiming to identify relationships between the structured assessment of the Achilles tendon and its function that could support injury prevention. We examined 72 athletes to investigate the link between Achilles tendon structure, as visualised in magnetic resonance images using a precise T2*-weighted gradient echo sequence with very short echo times, and its functional attributes. The acquired data were analysed using advanced artificial intelligence techniques and reviewed by radiologists. Additionally, we conducted statistical assessments to explore relationships with functional studies in four meaningful groups: dynamic strength, range of motion, muscle torque and stabilography.

**Results:**

The results show notable linear or non-linear relationships between functional indicators and structural alterations (maximal obtained Spearman correlation coefficients ranged from 0.3 to 0.36 for radiological assessment and from 0.33 to 0.49 for artificial intelligence assessment, while maximal normalised mutual information ranged from 0.52 to 0.57 for radiological assessment and from 0.42 to 0.6 for artificial intelligence assessment). Moreover, when artificial intelligence-based magnetic resonance assessment was utilised as an input, the associations consistently proved more robust, or the count of significant relationships surpassed that derived from radiological assessment. Ultimately, utilising only structural parameters as inputs enabled us to explain up to 59% of the variance within specific functional groups.

**Conclusions:**

This analysis revealed that structural parameters influence four key functional aspects related to the Achilles tendon. Furthermore, we found that relying solely on subjective radiologist opinions limited our ability to reason effectively, in contrast to the structured artificial intelligence assessment.

**Study Design:**

Cross-sectional studies.

**Supplementary Information:**

The online version contains supplementary material available at 10.1186/s40798-024-00786-6.

## Key Points

•Establishment of a relationship between magnetic resonance imaging-based structural features and functional characteristics of the Achilles tendon, emphasising non-linear connections.

•Introduction of a fast and efficient magnetic resonance imaging protocol for concurrent evaluation of structural and functional aspects, alongside a comparative analysis of artificial intelligence and radiologist reasoning capabilities.

•Highlighting the advantages of artificial intelligence-driven reasoning in identifying structural biomarkers, offering potential enhancements for injury prevention strategies and personalised treatment approaches.

**Supplementary Information:**

The online version contains supplementary material available at 10.1186/s40798-024-00786-6.

## Background

The Achilles tendon (AT) is the largest and strongest tendon in the human body, and it plays a crucial role in the musculoskeletal system. It facilitates everyday movements by withstanding high loads and storing energy, particularly during sports activities. There is a significant association between Achilles tendon dysfunctions and lower leg injuries. In addition, tendon disorders comprise 30 to 50% of all sports-related injuries [1]. Detecting and responding to developing pathologies within the tendon structure at an early stage can help reduce injuries and introduce significant cost savings, particularly in sports medicine.

The study by Parekh and Shah [2] shows that 26% of National Football League (NFL) players with Achilles tendon ruptures never returned to play. In the National Basketball Association (NBA), post-injury, 36.8% either retired or played fewer than 10 games [3]. Among British Rugby Union players, Achilles injuries are the third leading cause of missing over 100 days [4]. Additionally, half of distance runners and many triathletes suffer from Achilles tendinopathy [5, 6]. The incidence of these injuries has surged tenfold in 30 years [7, 8], placing a considerable financial strain on the healthcare system [9–11]. This underscores the need for early detection and prevention of Achilles tendon issues [12].

Predicting Achilles Tendon Injury (ATI) symptoms is central to many studies. Research by Mahieu et al. [13] identified plantar flexors’ strength and dorsiflexion range as key ATI predictors. O’Neill’s 2019 study corroborated this with similar findings in runners [14]. Studies with volleyball players [15, 16] suggested muscle power asymmetry during single-leg vertical jumps as another indicator. Additionally, ATI risk is associated with factors like genetics, nutrition, and training [17–19].

Many of the aforementioned factors have been assessed using medical imaging techniques such as Ultrasonography [20, 21] and Magnetic Resonance Imaging (MRI) [22], but not yet with artificial intelligence (AI) analysis. Ultrasound, being more accessible and cost-effective, is preferred over MRI. Some studies suggest that ultrasound and MRI are similarly effective in diagnosing tendinopathy [23, 24]. MRI, however, excels in operator independence and structural analysis.

Using Short or Ultrashort Echo Time (UTE) and T2*GRE (Gradient Echo) MRI techniques improves early Achilles tendon pathology detection [25]. These methods increase the visibility of minor tissue changes, making small lesions and irregularities more detectable. Combining these with structured descriptions and AI algorithms enhances precise MRI image interpretation, aiding in identifying biomarkers for musculoskeletal dysfunctions related to the Achilles tendon. Understanding the tendon’s structure is crucial for studying its responses to stress, injury, and targeted Achilles disorder treatments.

Our study primarily aims to link the observed structural features using MRI sequences characterised by short echo time to the Achilles tendon’s functional characteristics. This effort leads to the introduction of a novel, quick, and cost-effective protocol for monitoring the tendon’s structural and functional aspects. We’re considering factors like dynamic strength, stabilographic indices, range of motion, and muscle torque as important markers for assessing the Achilles tendon’s health and performance. Additionally, we aim to compare AI’s reasoning with radiologists’ in the context of our methodology, reflecting growing interest in this area.

## Methods

### Participants and Inclusion Criteria

**Fig. 1:**
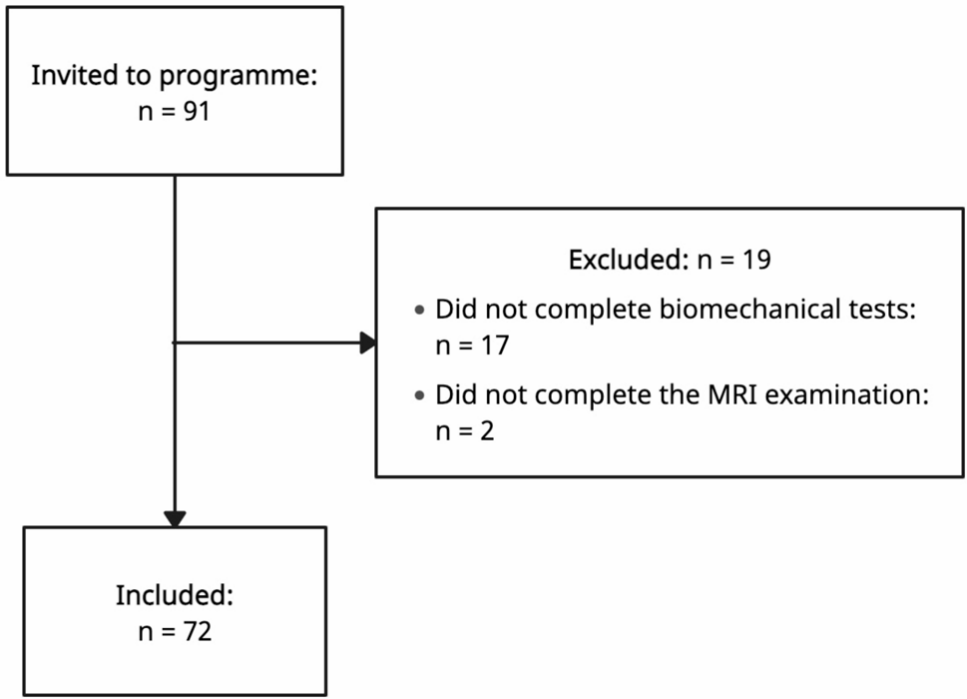
Flowchart of the participants’ inclusion process

Seventy-two sports individuals were selected (Fig. 1), meeting criteria including regular physical activity - at least two training sessions weekly in team sports with common Achilles Tendon Injuries (ATIs) like Rugby, Volleyball, Soccer, Badminton, and individual sports such as rowing, where the risk of ATI is linked to general training rather than the specific demands of the sport (Table 1). The training activities involve running or lower limb gym sessions, comprising more than 30% of the athletes’ total yearly training volume. All of them without ATIs history. Exclusion criteria included contraindications for MRI, acute lower limb injuries, and exacerbations of other illnesses.

**Table 1:** Demographic characteristics of the participants

Sex	Sport	*n*	Age [years]	Height [cm]	Body mass [kg]	Soft tissues mass [kg]	Fat content [%]
Man	Individual sports	19 (5 martial arts, 4 gym, 3 rowing, 2 badminton, 2 cycling, 2 tennis, 1 speed skating)	38.1 ± 9.7	183.1 ± 6.1	89.7 ± 11.2	66.7 ± 8.4	19.8 ± 7.5
	Team sports	26 (9 basketball, 9 rugby, 7 volleyball, 1 football)	36.9 ± 9.7	181.9 ± 7.6	84.9 ± 12.5	62.9 ± 8.5	19.8 ± 4.2
	Total	45	37.4 ± 9.6	182.4 ± 7.0	86. ± 12.1	64.5 ± 8.6	19.8 ± 5.7
Woman	Individual sports	10 (2 badminton, 2 rowing, 2 tennis, 4 gym)	37.0 ± 5.4	166.1 ± 5.7	63.5 ± 7.5	42.7 ± 2.7	26.4 ± 6.4
	Team sports	17 (9 Rugby. 2 handball, 3 volleyball, 2 football, 1 basketball)	25.7 ± 7.1	170.8 ± 7.8	67.5 ± 15.5	46.1 ± 7.1	24.8 ± 6.9
	Total	27	29.9 ± 8.5	169.0 ± 7.3	66.0 ± 13.1	44.8 ± 6.0	25.4 ± 6.7

### Procedures

The study protocol consisted of two main components: MR imaging and functional assessments. Data collection spanned from March through November 2022. Functional tests were conducted within a specialised biomechanical laboratory, while MRI tests were performed at a medical imaging facility. To minimise the time gap between the MRI scans and functional assessments, over 90% of the participants underwent both studies on the same day. For the remaining participants, the maximum time gap between the two studies was one week. None of the participants reported any injuries or issues with their Achilles tendon between the MRI and functional assessments.

#### MRI Assessment

Short MRI scans of both lower legs were performed with the use of two MRI scanners: the 3T machine (Siemens MAGNETOM Vida; Siemens Healthineers, Erlangen, Germany) and the 1.5T machine (United Imaging Healthcare uMR 588; United Imaging Healthcare, Shanghai, China). The whole protocol consisted of T2* GRE Echo sequence (with a short echo time ranging from 3.51 to 4.7 ms) acquired in the axial plane using Foot/Ankle coil type. Imaging parameters included a slice thickness of 3 mm, slice spacing from 2.48 mm to 4.19 mm, repetition times of 500 ms or 567.2 ms, and a field of view (FOV) ranging from 120 × 120 mm to 170 × 170 mm. The patient was positioned feet-first, supine (FFS). Scans were taken from the heel bone to the midpoint of the lower leg.

A structured description following Kapinski et al. model was used [26]. It includes 6 parameters describing the condition of the Achilles tendon:

1. Structural changes within the tendon (SCT) - informs about the loss of cohesion within the tendon area.
2. Tendon thickening (TT) - informs about the maximum dimension in the sagittal direction.
3. Sharpness of the tendon edges (STE) - informs about the edge fractality.
4. Tendon oedema (TE) - informs about an abnormal accumulation of fluid in the interstitium of the tendon.
5. Tendon uniformity (TU) - informs about the level of similarity of subsequent cross-sections of the tendon.
6. Tissue oedema (TisE) - informs about an abnormal accumulation of fluid and the enlarged size of the fascial compartment.

An experienced musculoskeletal radiologist with over 20 years of expertise in the field performed the assessment, with a total of 12 parameters assessed (6 for each leg). For each of the parameters, the expert could select a single score on an 8 point scale, where 0 describes a healthy tendon and 7 describes a severely injured one. Additionally, on the same scale, inference was performed using the SmarterOrthoMRI software (Smarter Diagnostics Sp. z o.o., Warsaw, Poland) [27] for comparison.

#### Functional Assessment

Functional studies involved body composition, plantarflexion and dorsiflexion isometric strength and range of movement (ROM), static balance parameters, power of the lower extremities, and the height of the rise of the body mass centre (COM) during a series of vertical jumps. All measurements were conducted on both lower limbs and repeated twice. The better result from the two repetitions of each functional assessment test was chosen for analysis. The functional variables were divided into four groups: dynamic strength, stabilographic indices, range of motion (ROM), and muscle torque. The biomechanical measurements were carried out in a specialised biomechanical laboratory, using certified equipment and were supervised by an experienced researcher who ensured the proper performance of the tests. The full results of functional tests are attached as supplementary material.

Dynamic strength was assessed by measuring lower extremity power and body mass centre (COM) rise during vertical jumps on a force plate (JBA Zb. Staniak, Warsaw, Poland). Participants executed eight jumps, two of each kind: Counter-movement jump (CMJ), single-leg counter-movement jump (SCMJ), and squat jump (SJ) [28]. For further calculations, dynamic strength parameters related to CMJ, SCMJ and SJ such as maximum force, maximum speed, maximum power, average power, total work, jump height calculated from speed, jump height calculated from flight time, jump height calculated from the work, jump height calculated from the trajectory of the centre of mass, swing depth, torque during take-off, time from the start of the movement to take-off, derivative of maximum force, time from reaching maximum force to take-off, derivative of maximum power and time from reaching maximum power to take-off, were taken.

Stabilographic indices were evaluated using the AMTI AccuSwayPLUS Balance Platform (ACS Model; Toronto, ON, Canada) through four trials (two per leg) involving one leg stance with eyes open and closed, each lasting 10 s and repeated twice [29]. The study included parameters related to displacement area and the path of the centre of mass.

Range of motion (ROM) was measured with a Seahan handheld goniometer in a prone position, focusing on the knee and ankle’s passive and active ROM, plus their differences and ratios [30].

Muscle torque for plantarflexion and dorsiflexion was measured using a specialised device (JBA Zb. Staniak, Warsaw, Poland) in a seated position, following the method described by Konik et al. [31]. The participants were positioned with hips and knees at 90°, arms crossed on the chest and ankles in a neutral position.

### Statistical Analysis

The Mann-Whitney U test was employed to compare MRI assessments made by the radiologist and the AI. Results with a p-value < 0.05 were considered statistically significant. Mean absolute errors (MAE) were calculated for each of the six structural parameters to illustrate the discrepancies between the radiologist’s and AI’s assessments. The mean values ​​of each parameter for AI and radiologist assessments are also reported.

The study analysed 100 parameters from functional studies: 72 from dynamic strength, 8 from stabilographic indices, 16 from range of motion, and 4 from muscle torque. Patients who did not undergo at least one examination had their results excluded from the analysis. However, this exclusion was specific to the stabilography tests, affecting 28 patients. Principal Component Analysis (PCA) was conducted to reduce these parameters while maintaining information integrity. The top 8, 5, 5, and 2 PCA parameters for each group, explaining over 90% of the variance, were selected as meta-parameters.

Linear and non-linear relationships between structural and functional parameters were explored using Spearman correlation and normalised mutual information (NMI), as they are common metrics used for presenting both types of relationships that were expected between structural and functional data. These measures were calculated between structural features and functional meta-parameters for each category, both for radiologist-assessed and AI-assessed (by SmarterOrthoMRI software) parameters. Statistical significance was established with p-values under 0.05 for 95% confidence. For NMI, a 30% threshold was used [32]. This data created a list of features most common in significant relationships.

The feature importance was utilised to design a transformation function that explained the most variance in function based on AT structural parameters. Linear optimisation, based on the Powell method, was attempted using features initiated from the importance list with optimised weights. Non-linear models, i.e. Decision Trees [33], Random Forests [34], and ensembles with the AutoGluon [35] library, were employed to capture more complex relationships.

The statistical analysis and presentation are consistent with the Checklist for Statistical Assessment of Medical Papers statement [36].

### Machine Learning Analysis

For each group, an ensemble of models was created. These models were trained using structural features to predict each of the selected functional meta-parameters (PCA components). The objective was to achieve the highest explained variance score for the entire functional group, based on structural features labelled by both an AI model and a radiologist. Due to the limited sample count, the AI models were trained using a 10-fold cross-validation approach.

The initial step involved training Decision Tree and Random Forest models. The best model was selected based on its *r*^2^ score on the given test set and the whole dataset. This metric was chosen due to its similarity to the explained variance coefficient.

The second approach was to use the AutoGluon package. During this process, we employed Bayesian optimisation with a fixed number of trials to find a model with the highest *r*^2^ score.

For the models that explained the most total variance in the four functional groups, a list of the structural parameters that had the most significant impact on the model was extracted. In the case of machine learning (ML) models, these features were selected by calculating permutation importance [34] and normalising it to a 0–1 range. This analysis provided insights into how specific structural parameters contributed to model reasoning and their relationship with function.

### Patient and Public Involvement

There was no patient or public involvement in the planning, conceptualization, research design, analysis, interpretation, or composition of the findings. However, every patient was instructed on the clinical value of the tests performed and could request individual results of the structural assessment and receive a report. Interested patients also took part in a seminar that introduced the research concept and concluded with a comprehensive discussion between the study participants and researchers.

### Equity, Diversity and Inclusion Statement

Our research team consists of seven men and three women from Europe. The study population is diverse in terms of age, gender, demographics, and comorbidities. However, the predominantly white composition of the cohort resulted in an underrepresentation of other ethnic groups. Individuals from marginalised communities may also have lower representation. Methodological constraints required excluding individuals with specific comorbidities and mobility limitations from analyses.

## Results

### MRI Assessment

**Fig. 2:**
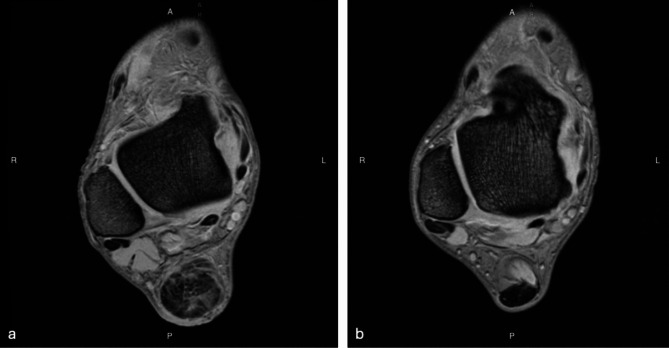
A pictorial comparison of images obtained using T2* GRE sequence with short echo time for patients with high (**a**) and low (**b**) values of structural parameters (indicating worse and better Achilles tendon conditions, respectively; labels: A – anterior; P - posterior, L – left; R -right)

The MRI examinations were assessed for six structural parameters of the Achilles tendon by both a radiologist and an AI model (Table 2; Fig. 2). There were no statistically significant differences between the radiologist and AI assessments. The average MAE values were within the range of 0.23–0.60.

**Table 2:** Comparison of AI and radiologist structural assessments *SD (standard deviation)

parameter	leg	mean MAE ± SD	mean value ± SD - radiologist	mean value ± SD - AI	significance of difference (radiologist vs. AI)	*p*-value
SCT	left (*n* = 72)	0.6 ± 0.82	1.65 ± 1.27	1.47 ± 0.53	not significant	0.782485
	right (*n* = 72)	0.54 ± 0.69	1.71 ± 1.13	1.53 ± 0.58	not significant	0.645344
	total (*n* = 144)	0.57 ± 0.75	1.68 ± 1.20	1.5 ± 0.56	not significant	0.882789
TT	left (*n* = 72)	0.26 ± 0.65	0.99 ± 0.97	0.94 ± 0.87	not significant	0.934106
	right (*n* = 72)	0.23 ± 0.46	1.04 ± 1.05	1.0 ± 1.02	not significant	0.822369
	total (*n* = 144)	0.25 ± 0.55	1.01 ± 1.01	0.97 ± 0.95	not significant	0.834690
STE	left (*n* = 72)	0.47 ± 0.58	0.93 ± 0.76	1.01 ± 0.12	not significant	0.089506
	right (*n* = 72)	0.4 ± 0.66	1.14 ± 0.83	1.01 ± 0.12	not significant	0.626514
	total (*n* = 144)	0.44 ± 0.62	1.03 ± 0.79	1.01 ± 0.12	not significant	0.343004
TE	left (*n* = 72)	0.5 ± 0.69	0.92 ± 1.08	0.78 ± 0.81	not significant	0.738311
	right (*n* = 72)	0.6 ± 0.76	1.04 ± 1.19	0.86 ± 0.88	not significant	0.572972
	total (*n* = 144)	0.55 ± 0.73	0.98 ± 1.14	0.82 ± 0.84	not significant	0.529274
TU	left (*n* = 72)	0.29 ± 0.59	0.90 ± 0.97	0.69 ± 0.76	not significant	0.272347
	right (*n* = 72)	0.24 ± 0.42	0.99 ± 1.01	0.86 ± 0.94	not significant	0.400703
	total (*n* = 144)	0.26 ± 0.51	0.94 ± 0.99	0.78 ± 0.85	not significant	0.165372
TisE	left (*n* = 72)	0.44 ± 0.58	0.96 ± 0.96	1.04 ± 0.94	not significant	0.476398
	right (*n* = 72)	0.46 ± 0.58	1.14 ± 0.94	1.07 ± 0.94	not significant	0.864096
	total (*n* = 144)	0.45 ± 0.58	1.05 ± 0.95	1.06 ± 0.94	not significant	0.705424

MAE - mean absolute error; SCT – structural changes within the tendon; STE - sharpness of the tendon edges; TE – tendon oedema; TisE - tissue oedema; TT – tendon thickening, TU – tendon uniformity

### Functional Assessment

100 parameters from four functional groups - dynamic strength, stabilographic indices, range of motion, and muscle torque - were collected for analysis. The results of specific tests are included as an additional file alongside the manuscript (Additional File 1).

### Relationships - AI vs. Radiologist

**Fig. 3:**
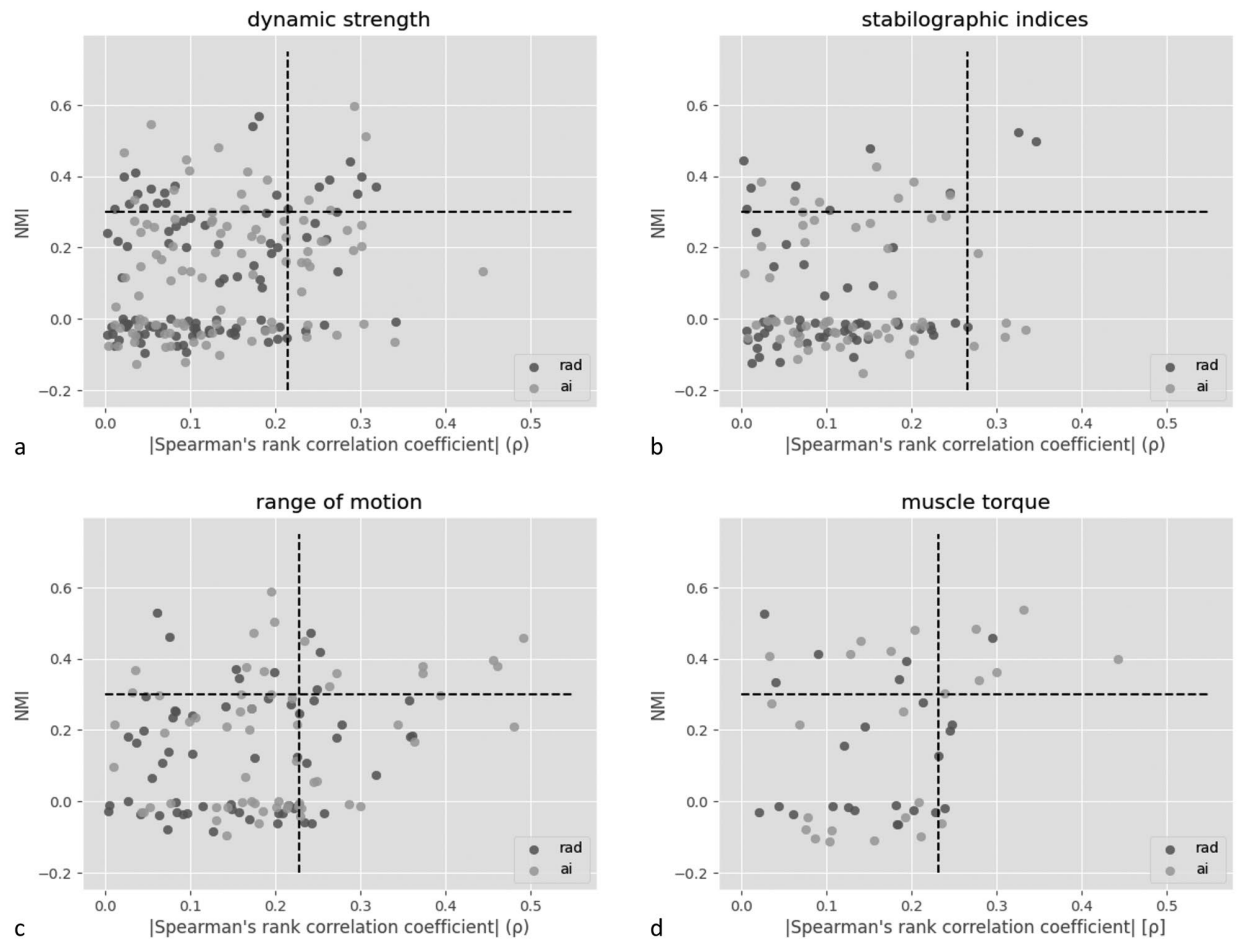
Values of Spearman correlation coefficients and normalised mutual information (NMI) between structural features indicated by both the radiologist (rad) and SmarterOrthoMRI (ai) software and functional meta-parameters related to dynamic strength (**a**), stabilographic indices (**b**), range of motion (**c**) and muscle torque (**d**)

The relationships were categorised according to the obtained values into 4 groups: high correlation coefficient and high mutual information, low correlation coefficient and high mutual information, high correlation coefficient and low mutual information, and low correlation coefficient and low mutual information (see Fig. 3).

Structural parameters with a significant correlation coefficient and normalised mutual information over 0.3 were used to create a list of the top significant features in each of the 4 categories, separately for features obtained from radiologists and the software.

**Fig. 4:**
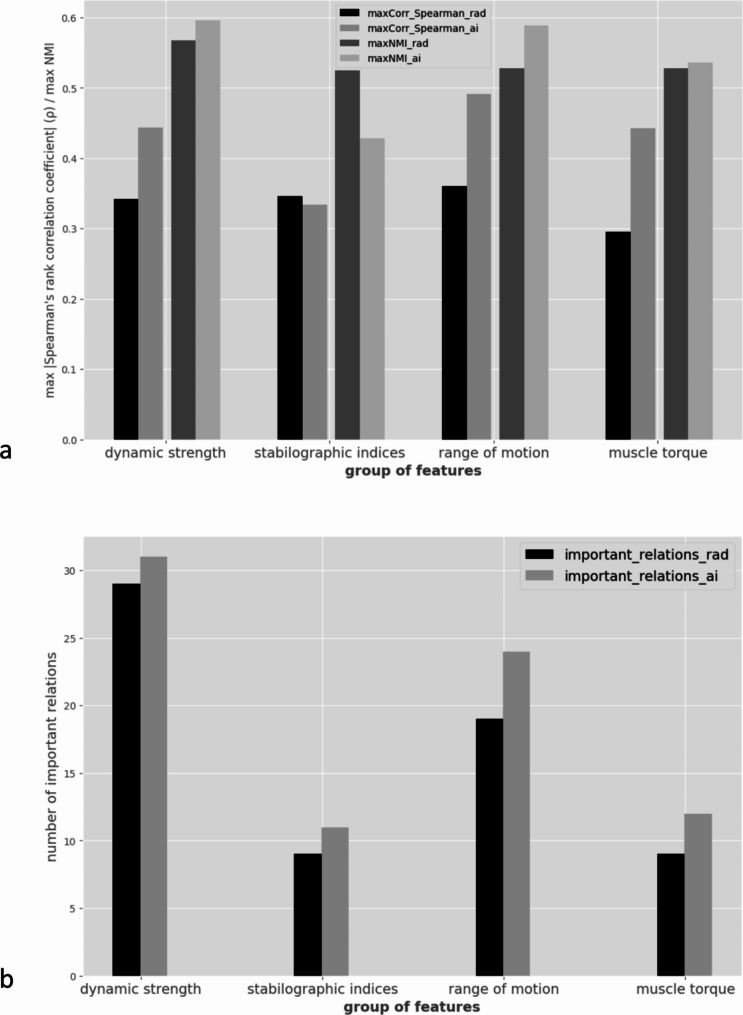
Comparison between the maximal values of Spearman correlation coefficients and normalised mutual information (**a**), as well as the total number of significant relationships (**b**) between structural features indicated by the radiologist (rad) and SmarterOrthoMRI software (a.i.) and functional meta-parameters in four different groups

For 3 out of 4 categories of functional features, the maximum obtained correlation coefficients and normalised mutual information were higher for calculations using structural features obtained from software evaluation than those assessed by a radiologist (Fig. 4a; Table 3). Only in the case of functional features related to stability, higher results were obtained for radiological assessments.

**Table 3:** Comparison of the relationships between achilles tendon structure assessed by radiologist and AI and functional meta-parameters

	Max spearman correlation coefficients		Max normalised mutual information (NMI)		Total number of significant relationships (structure vs. function)	
group of features	radiologist assessments	AI assessments	radiologist assessments	AI assessments	radiologist assessments	AI assessments
dynamic strength	0.34	0.44	0.57	0.6	29	31
stabilographic indices	0.35	0.33	0.52	0.42	9	11
range of motion	0.36	0.49	0.53	0.59	19	22
muscle torque	0.3	0.44	0.53	0.54	9	12

The number of all significant structure-function relationships was summed up. Significant relationship was understood as one where at least one of the examined metrics (correlation coefficient or normalised mutual information) was above the assumed significance threshold. In 3 out of 4 categories of functional features groups, their number was greater for the study using structure assessments from the AI software than those from the radiologist (Fig. 4b; Table 3). Only in the case of functional features related to muscle torque a greater number of significant relationships were obtained for radiological assessments.

### Transformation Function

**Fig. 5:**
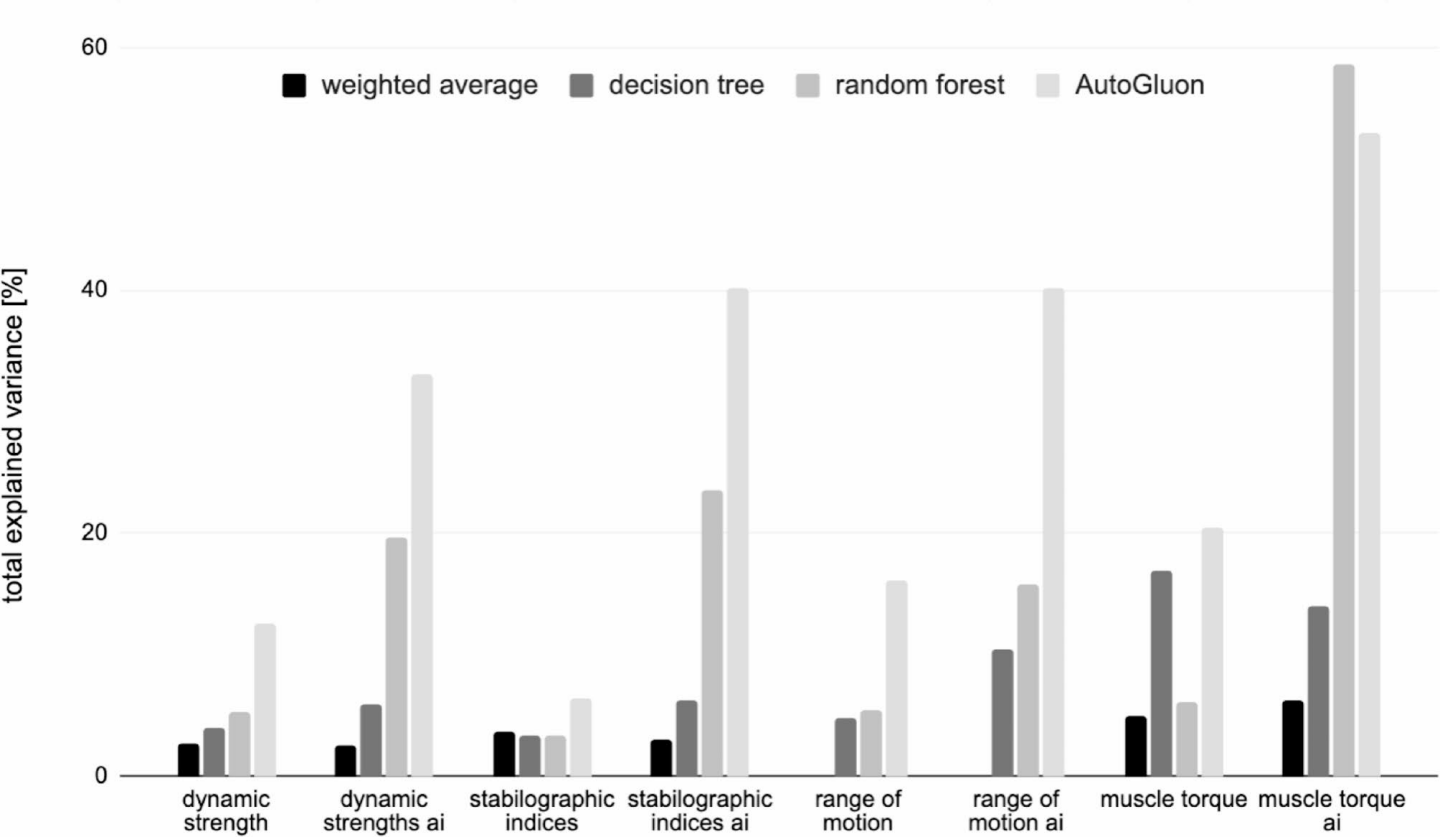
Level of total variance of functional meta-parameters explained by structural features being input to different types of transformation functions: decision trees, random forest, AutoGluon models and weighted average with optimised weights

Four approaches were used to find the best transformation function of the structural information in order to explain the most variance in the functional data. In 7 out of 8 tested cases, AutoGluon models coped best with this task. In one case, i.e. features related to muscle torque, the random forest model performed best, explaining 59% of the variance (Fig. 5).

In all groups of functional features, the best results were achieved for relationships with the assessments from AI models (Table 4). The explanation of linear relationships using the weighted average of structural features with weights selected in the optimisation process did not exceed 7% of the explained variance of functional features, indicating that there are rather non-linear relationships between function and structure.

**Table 4:** Total variance explained by the best model

Group of features	Radiologist assessments	AI assessments
dynamic strength	13%	33%
stabilographic indices	6%	40%
range of motion	16%	40%
muscle torque	20%	59%

### Achilles Tendon Structural Biomarkers

**Fig. 6:**
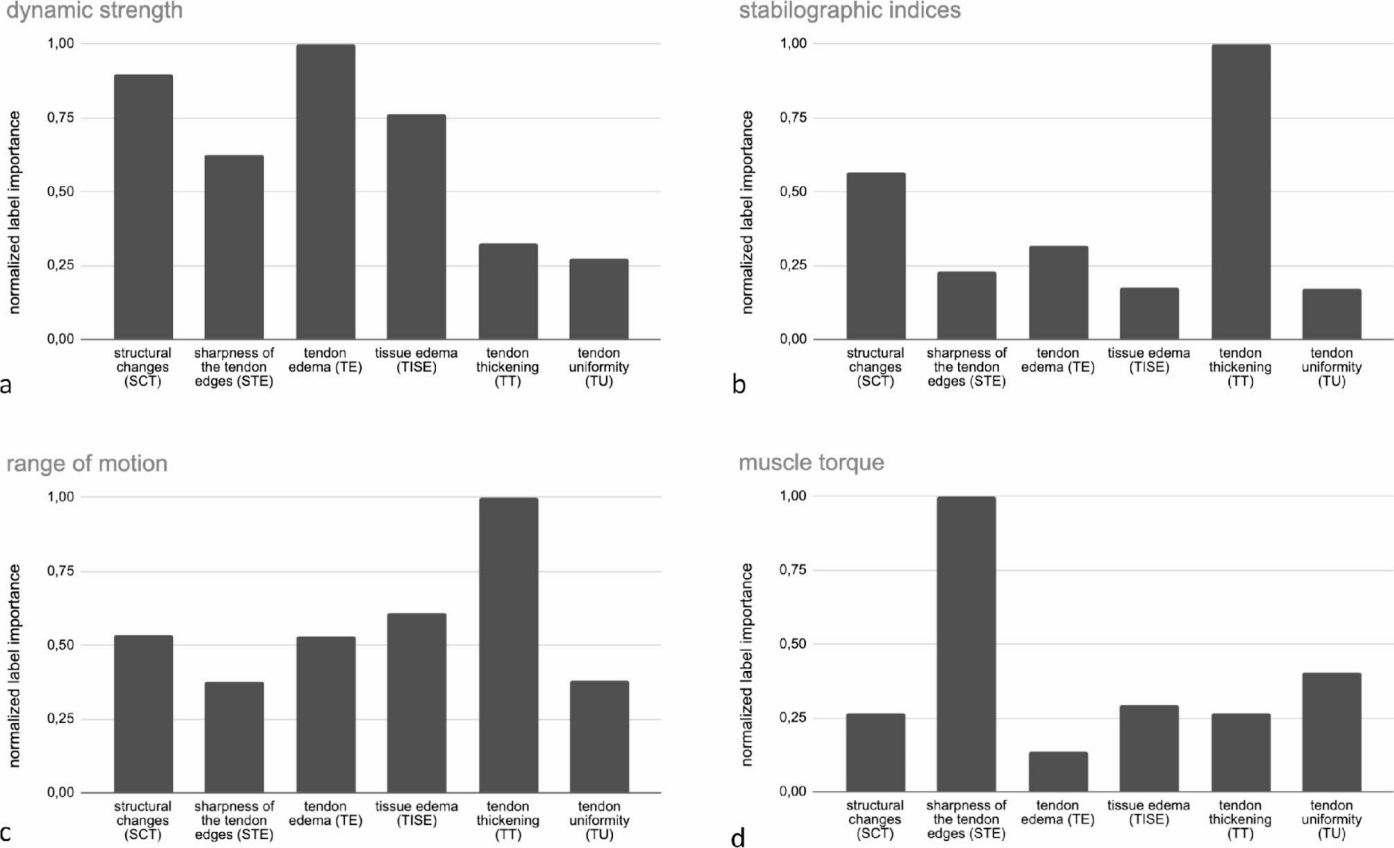
Normalised contribution of individual structural features in explaining the variance of functional meta-parameters related to dynamic strength (**a**), stabilographic indices (**b**), range of motion (**c**) and muscle torque (**d**)

For each set of features, the best model used different structural features with different importance to predict the result. The normalised significance of features is shown in the following figures: dynamic strength (Fig. 6a), stabilographic indices (Fig. 6b), range of motion (Fig. 6c), and muscle torque (Fig. 6d).

The most important contribution to the dynamic strength relationship was made by tendon oedema, followed by structural changes. In the case of the stabilographic indices, the most important feature was tendon thickening, followed again by structural changes. For range of motion, tendon thickening and tissue oedema were the most significant factors. In the case of muscle torque, we observed the highest contribution from the sharpness of tendon edges and tendon uniformity. Although tendon thickening appeared in first place in two cases, i.e. stabilographic indices and range of motion, the unique combination of parameters contributions constituted by at least two structural factors appeared in all studied functional groups allowing for further work on unique biomarkers definition.

## Discussion

In this study, we proposed a novel AI and MRI-based protocol to examine the relationship between the structural aspects of the Achilles tendon and functional metrics in sports participants. Our investigation encompassed both linear and non-linear methodologies, a comparison between AI-driven reasoning and radiologist assessments, and an exploration of the significance of recognising structural biomarkers. These biomarkers could potentially be applicable for injured patients undergoing medical surgeries, checkups, and patient recovery, as well as for the context of healthy individuals to supervise athletes, reduce injuries, and apply personalised treatment tactics in professional sports. Moreover, the protocol consists of both leg examinations, is cost-effective, as it is based on a very short gradient sequence, with an echo time less than 5 ms, and results in a participant turnaround time of less than 15 min. While sequences like Proton Density Fat Sat are commonly used in musculoskeletal assessments, we opted for the T2* GRE sequence with a short echo time due to its enhanced sensitivity in detecting subtle tendon changes, as supported by both existing literature [37] and our comparative studies. This fact implies the possibility of using it as a population screening tool for the early detection of structural and functional risk factors, which could lead to the implementation of early treatment tactics and a reduction in the number of injuries, thus introducing significant cost savings for stakeholders.

Literature shows a link between tendon structure and function, like diverse tenocyte responses to different types of mechanical stress [38, 39]. Our findings on the Achilles tendon reveal both linear and non-linear relationships between these aspects, highlighting their complex nature. This underscores the need for non-linear methods when investigating the influence of the Achilles tendon on functional results. Our research complements the studies by Szaro et al. [22], which examined geometric differences in Achilles tendon dimensions and their correlations in normal and tendinopathic tendons using MRI. As the authors mentioned, the dimensions of the Achilles tendon impact its function: a longer tendon enhances endurance running performance, while an increased diameter is associated with degeneration, reduced stiffness, and lower tensile strength. Our studies further this understanding by mapping AT structure to its function and illustrating the nature of these relationships.

Our study found AI-driven descriptions more effective than radiologist evaluations in identifying important relationships and explaining variance in functional data (33–59% with AI, 6–20% with radiologists). The given outcomes may result from the fact that in regular settings, radiologists utilise more than just one MRI sequence to assess the tendon, thus making it not only more time-consuming but also more accurate. It allows them to cross-verify findings, reducing the likelihood of missing subtle changes in the tendon tissue, but also relies heavily on the radiologist’s experience and expertise. The integration of AI algorithms, with their capacity to process substantial datasets and exhibit generalisation, enabled a way to limit the number of sequences and make more thorough and precise evaluations of structural as well as functional aspects within one examination. This highlights the trade-offs between the comprehensive, yet subjective and time-consuming nature of radiologist evaluations, and the efficient, consistent, and objective assessments provided by AI. By providing this context, we aim to emphasise the complementary roles of radiologists and AI in MRI evaluations and how they can be integrated to enhance the accuracy and efficiency of Achilles tendon injury assessments. The results align with other research using AI in radiology [40–43] and sports injury prediction [44, 45], indicating AI’s growing role in enhancing diagnostic accuracy and treatment strategies in musculoskeletal health.

Our research also delved into structural biomarkers. By identifying key structural parameters that strongly influenced models explaining functional differences, we uncovered potential markers for injury risk monitoring. Detecting early pathologies that affect function can guide personalised approaches such as nutritional adjustments, training modifications, and supplementation to reduce injury risks [46]. Furthermore, understanding the connection between functional issues and underlying structure is essential for minimising time away from activities. This knowledge aids in selecting targeted treatment strategies that address individual needs based on a short MRI examination.

### Clinical Implications

Objective AI-based assessment of MRI biomarkers within the T2* Gradient Echo sequence with short echo time is crucial for early detection of structural changes in collagen tissue, such as the Achilles tendon, and can be integrated into clinical practice as a screening tool, post-surgery assessment, return-to-sport protocols, and in developing novel treatments. The identified connections between the Achilles tendon structure and its function may offer valuable perspectives for healthcare professionals, especially those in sports clubs and academia, assisting in the evaluation of elements associated with Achilles tendon injuries. The use of AI in assessments holds the promise of improving orthopaedic procedures by accelerating patient turnover through swift medical image analysis. It also introduces a pioneering and thorough structured analysis well-suited for injury reduction strategies.

### Limitations

While our study contributes valuable insights into the relationship between Achilles tendon structure and function, it is important to acknowledge some limitations. The sample size of our study was relatively small, and further studies with larger cohorts are needed to validate our findings. Additionally, the use of AI algorithms and machine learning techniques relies heavily on the quality and quantity of available data, and ongoing advancements in these areas may yield even more accurate and refined results in the future.

## Conclusion

In conclusion, our study highlights the non-linear nature of the relationship between Achilles tendon structure and function, while also introducing a short and cost-effective MRI-based protocol for both structural and functional assessment. The superiority of AI-based reasoning and the identification of structural biomarkers emphasise the potential for enhancing injury prevention strategies and customising treatments to individual needs. Future research should focus on expanding our understanding of the complex interactions between structure and function, as well as harnessing the power of AI and machine learning to optimise diagnostic and therapeutic approaches in orthopaedic settings.

## Electronic Supplementary Material

Below is the link to the electronic supplementary material.

Supplementary Material 1

## Data Availability

Data may be obtained after seeking approval from a third party (from Józef Piłsudski University of Physical Education in Warsaw and from the Institute of Sport - National Research Institute in Warsaw) and are not publicly available. The code of the software used for the study is not publicly available.

## Abbreviations

AI: Artificial intelligence
AT: Achilles Tendon
ATI: Achilles Tendon Injury
ATIs: Achilles Tendon Injuries
COM: Body mass centre
CMJ: Counter–movement jump
FOV: Field of view
GRE: Gradient Echo
MAE: Mean absolute error
ML: Machine learning
MR: Magnetic resonance
MRI: Magnetic resonance imaging
NBA: National Basketball Association
NFL: National Football League
NMI: Normalised mutual information
PCA: Principal component analysis
Rad: Radiologist
ROM: Range of movement
SCT: Structural changes within the tendon
SCMJ: Single–leg counter–movement jump
SD: Standard deviation
SJ: Squat jump
STE: Sharpness of the tendon edges
TE: Tendon oedema
TisE: Tissue oedema
TT: Tendon thickening
TU: Tendon uniformity
UTE: Ultrashort Echo Time
